# Sciatic nerve entrapment after total hip arthroplasty in a patient with diffuse lipomatosis and developmental dysplasia of the hip: A rare case report^☆^

**DOI:** 10.1016/j.ijscr.2024.110707

**Published:** 2024-12-04

**Authors:** Cai Jian, Zhou Guanming, Guan Mingqiang, Zeng Huiliang

**Affiliations:** Foshan Hospital of Traditional Chinese Medicine, China

**Keywords:** Hip dysplasia, Diffuse lipomatosis, Hip arthroplasty, Nerve damage；case report

## Abstract

**Introduction and importance:**

Diffuse lipomatosis is rare. In this case, a patient with diffuse lipomatosis and developmental dysplasia of the hip (DDH) underwent total hip arthroplasty (THA). Postoperatively, the patient experienced sciatic nerve entrapment. The lack of treatment experience and related literature makes addressing this complication challenging.

**Case presentation:**

A 26-year-old female patient presented to our clinic with developmental dysplasia of the right hip and lower limb malformations. She underwent THA and, one week later, developed contralateral sciatic nerve entrapment, for which she received neurolysis. The patient was diagnosed with diffuse lipomatosis based on pathological results. Four years post-operation, she lives independently and is satisfied with the treatment outcomes.

**Clinical discussion:**

This case report aims to illuminate the approach to diagnosing and treating patients with diffuse lipomatosis and DDH. By exploring the elements of this patient's journey to diagnosis and treatment, we aspire to aid future clinicians in navigating the challenges of performing THA for patients with anatomical abnormalities around the hip joint. Once neurological symptoms appear, sciatic nerve entrapment must be considered.

**Conclusion:**

Neurological symptoms in the lower limbs following THA in patients with diffuse lipomatosis should prompt consideration of sciatic nerve entrapment. Adjustments in body positioning may be an effective method to prevent nerve entrapment in these patients. Once sciatic nerve entrapment is diagnosed, early surgery neurolysis may reduce the occurrence of sequelae.

## Highlights

• DDH patients with diffuse lipomatosis are rare, treatments and outcomes are poorly described in the literature.
• Larger and heavier lower limbs caused by various reasons not only increased the difficulty of hip arthroplasty, but also increased the risk of dislocation after operation.
• Neurological symptoms in the lower limbs after THA in diffuse lipomatosis patients should raise suspicion for sciatic nerve entrapment.

## 1 Introduction

Developmental dysplasia of the hip (DDH) is a common orthopedic condition. However, cases of DDH combined with diffuse lipomatosis are rarely reported. Diffuse lipomatosis is characterized by widespread infiltration of mature adipose tissue within the skin, subcutaneous tissues, and muscles, often affecting large portions of an extremity or the trunk [1]. In this patient, the larger and heavier lower limbs due to diffuse lipomatosis not only increased the complexity of surgery but also elevated the risk of hip dislocation. Due to the rarity of DDH patients with diffuse lipomatosis, treatments and outcomes are sparsely documented in the medical literature. This report aims to detail the diagnosis and treatment of a DDH patient with diffuse lipomatosis. The patient consented to publishing her data per the SCARE criteria [2].

## 2 Case presentation

**Fig. 1:**
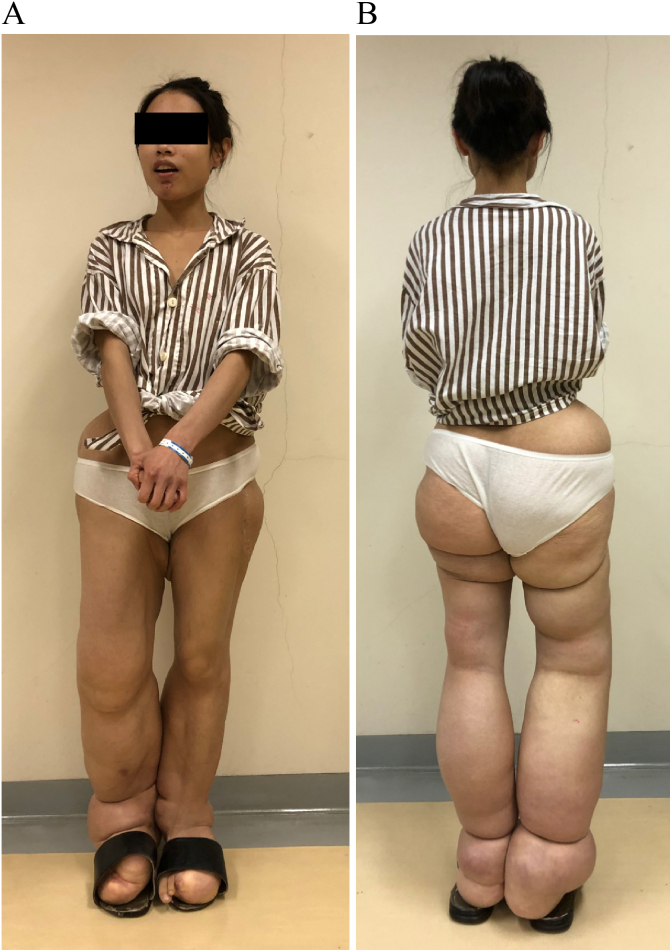
A 26-year-old woman with deformed lower limbs. She underwent four operations but could not improve.

**Fig. 2:**
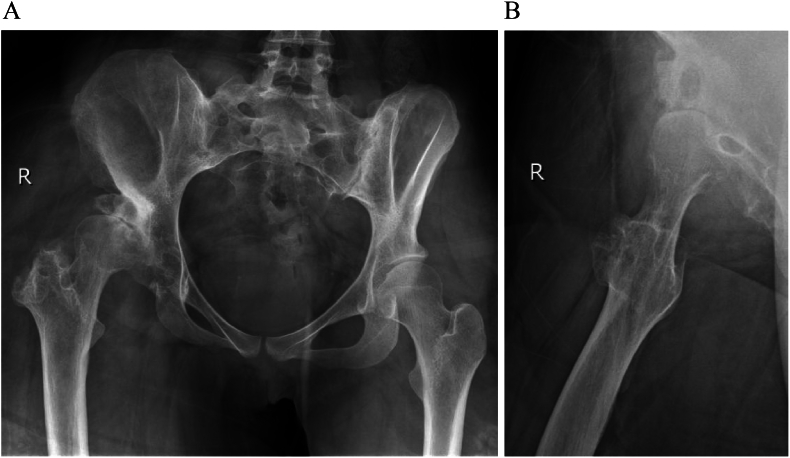
X-ray shows dysplasia and subluxation of the right hip (Crowe III).

**Fig. 3:**
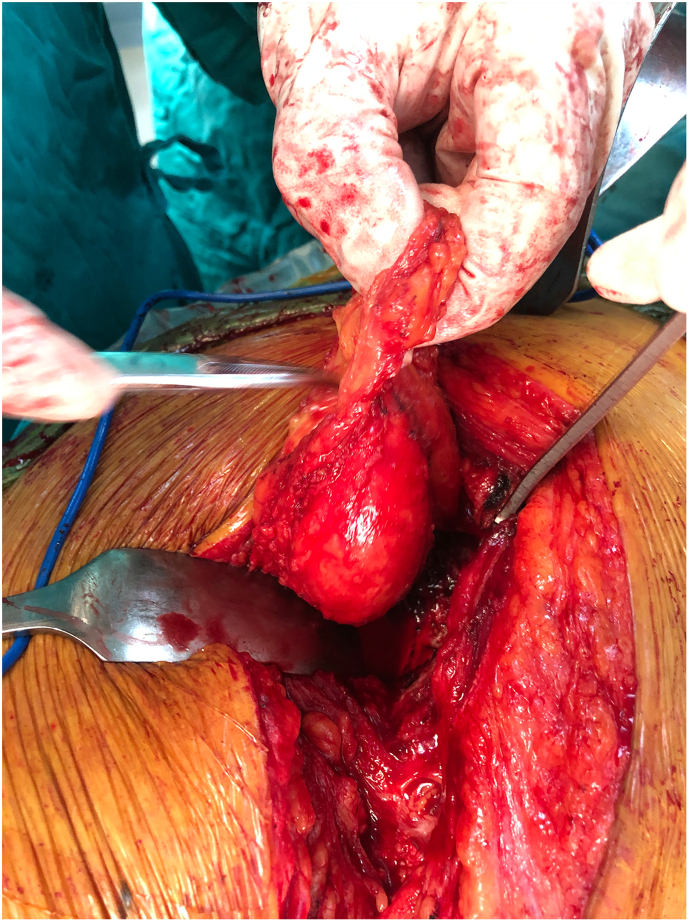
During THA, a large amount of adipose tissue around the joint was found, which was carefully separated and resected.

A 26-year-old female presented at our clinic with a three-month history of pain in her right hip. The patient denied any history of filariasis or relevant family medical issues. She reported being born with what she described as “rubber legs,” attributing this to her mother's use of Coldrex during pregnancy. She had undergone four operations on both lower limbs but could not provide specific details as she had lost the relevant medical records. Her condition had not progressed in recent years. Upon examination, both lower limbs appeared enlarged and deformed (Fig. 1), though muscle strength and sensation were normal. X-ray imaging identified dysplasia and subluxation of the right hip (Crowe III) (Fig. 2), and MRI scans showed fat accumulation in the buttocks. Color Doppler ultrasound and electromyography of the right lower limb showed no abnormalities. Given the severity of pain and the ineffectiveness of oral analgesics, a THA was performed on the right hip (using a Smith & Nephew total hip prosthesis). During the operation, a significant amount of adipose tissue around the joint was observed (Fig. 3), which was meticulously separated and excised.

After the operation, the patient experienced numbness and tingling below her left knee. Initially, it was suspected that the issue might originate from the lumbar region, but an MRI of the lumbar spine revealed no significant compression on the spinal cord or nerve roots. Subsequently, the patient reported severe pain in the left buttock and weakness in dorsiflexing the left ankle, suggesting a sciatic nerve injury. Ultrasound and MRI indicated compression and inflammatory changes in the left sciatic nerve at the gluteal segment (Fig. 5). Despite one week of treatment with intravenous methylprednisolone and mannitol, there was no notable improvement.

Surgery was performed using the K-L approach. A large amount of adipose tissue was removed, and the sciatic nerve was exposed, appearing swollen and congestive (Fig. 6). Most adipose tissue surrounding the sciatic nerve was excised, and an epineurotomy was performed for decompression. Postoperatively, the patient reported significant relief from pain. Within a week, she could walk with crutches, although she still could not dorsiflex the left ankle and toes. Pathology confirmed diffuse lipomatosis (Fig. 7).

After discharge, the patient continued to require crutches due to her inability to dorsiflex her left ankle and toes. The surgical incision healed well, without any signs of infection. Nine months post-operation, the patient could walk with a single crutch, employing increased knee flexion to compensate for the limited function of her foot and ankle, though she had to slow her movements to prevent falls. After one year, she could live independently but faced difficulties navigating stairs. She could travel to another province alone but relied on crutches for support. Four years post-operation, she reported no issues with her right hip but experienced pain in both ankles after extended periods of walking, with persistent inability to dorsiflex her left ankle and toes. Despite these challenges, she expressed satisfaction with the overall results of her treatment.

## 3 Discussion

Diffuse lipomatosis is rare, with scant literature available on the condition. Diagnosis primarily relies on pathological findings; family history and clinical manifestations are essential considerations. Its symptoms are similar to those of lower limb lymphedema, including swelling of the affected limb or region, typically described as soft and pitting [3]. In this case, the initial diagnosis in the outpatient department was lower limb lymphedema. However, this was ruled out as the patient's skin on her legs was normal, showing no signs of cutaneous or subcutaneous fibrosis. It is also crucial to differentiate diffuse lipomatosis from Madelung's disease or multiple symmetric lipomatosis (MSL). MSL, a rare fat metabolism disorder, typically manifests as subcutaneous, unencapsulated fatty tissue overgrowth that is painless and primarily affects the head, neck, upper torso, and upper extremities [3]. However, in this patient, the lesion was confined to the lower limbs, which is inconsistent with MSL. The final diagnosis was confirmed as diffuse lipomatosis through pathological evaluation.

Surgical intervention for diffuse lipomatosis is usually considered for cosmetic reasons or to prevent mechanical disturbances. However, it carries risks of complications such as extensive bleeding, vascular and nerve injuries, and infection [1,4]. In this case, despite the patient having undergone four operations and living with deformed lower limbs without pain or severe functional impairment for many years, the current hospital visit was prompted by hip pain rather than leg deformity. Therefore, we determined that further intervention for diffuse lipomatosis was unnecessary.

**Fig. 4:**
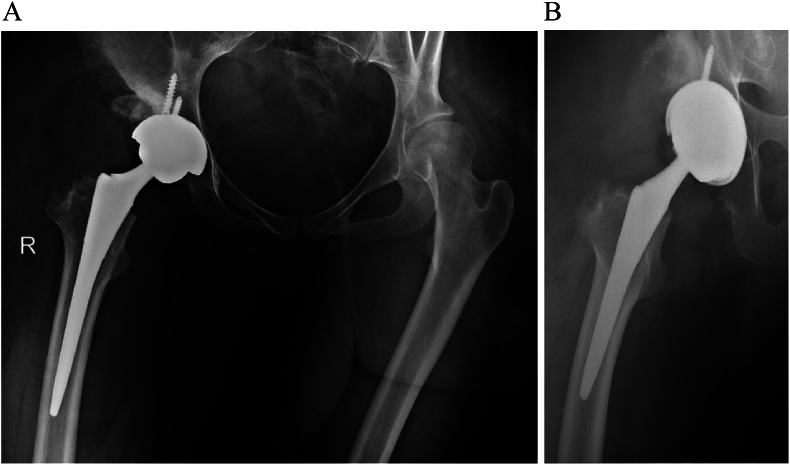
Abduction angle of the acetabulum cup was placed at about 35° to reduce the risk of dislocation.

To the best of our knowledge, there have been no reported cases of DDH combined with diffuse lipomatosis treated with hip arthroplasty. Vaishya et al. reported a case of DDH combined with lymphedema of the lower limbs, where the prosthesis was dislocated post-THA [5]. The authors speculated that the dislocation might have been due to the increased weight of the limb. In the present case, the muscle strength of the gluteus medius was evaluated pre-operatively through physical examination and electromyography. Given the patient's left-sided pelvic tilt during standing and walking, the abduction angle of the acetabular cup was set at approximately 35° to minimize the risk of dislocation (Fig. 4). Contrary to expectations, the patient developed contralateral sciatic nerve entrapment rather than hip dislocation post-operation.

**Fig. 5:**
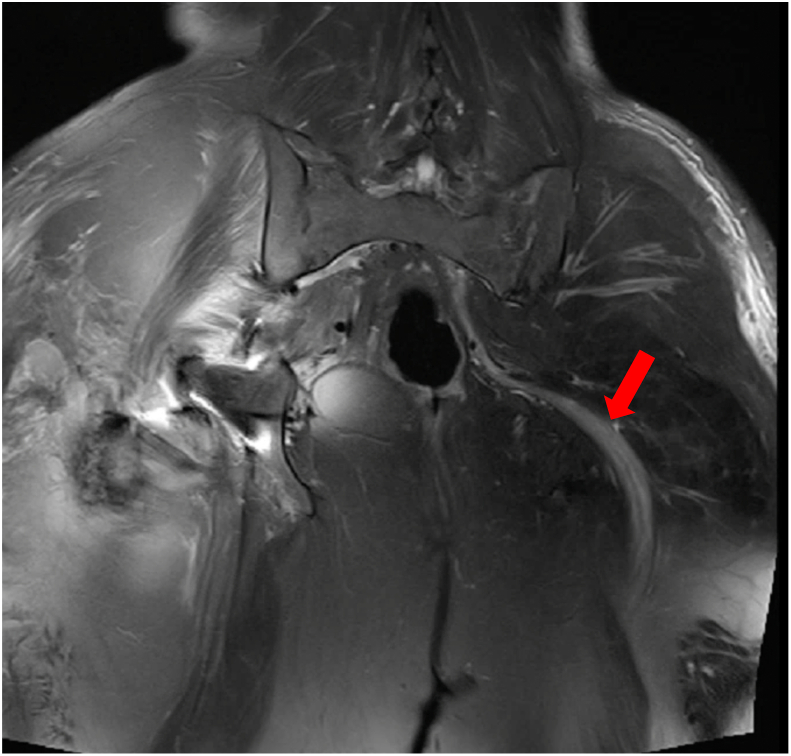
This MRI shows compression and inflammatory changes of the left sciatic nerve in the gluteal segment in T2WI.

**Fig. 6:**
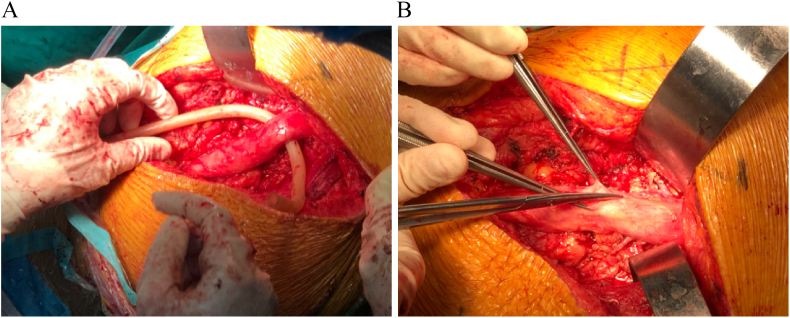
A. During the secondary operation, the sciatic nerve was dissected and separated, presenting swollen and congestive. B The epineurium of the sciatic nerve was incision to decompression.

**Fig. 7:**
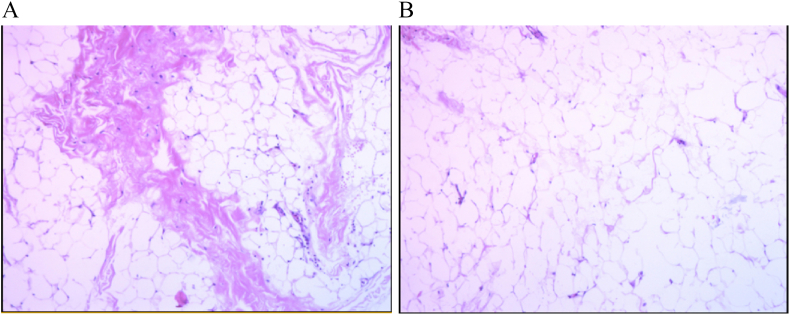
Under the microscope, many mature adipocytes and a small quantity of fibrous tissue can be seen.

Several factors may contribute to sciatic nerve entrapment, including piriformis hypertrophy, innominate band compression, abnormal sciatic nerve course, transverse vein compression, osteophyte proliferation, and gluteal muscle degeneration, all of which are associated with deep gluteal syndrome [6]. Although diffuse lipomatosis can cause hyperplasia of adipose tissue in subcutaneous tissue, fascia, and muscle [1,4,7], reports of it leading to sciatic nerve compression are scarce. Neurolipomatosis, where fat growth is confined to the nerve, presents similar symptoms but differs in that fat does not proliferate in surrounding tissues. According to pathological findings, nerve components can be identified in neurolipomatosis, unlike in diffuse lipomatosis [8]. The cause of the contralateral sciatic nerve entrapment remains unclear. We hypothesized that prolonged supine positioning due to the patient's heavy limbs post-surgery might have increased pressure on the buttock, leading to nerve entrapment. However, on the operated side, some adipose tissue was removed during THA, likely preventing nerve compression.

Neurolysis is recognized as a safe and effective treatment for sciatic nerve entrapment [9]. It can significantly alleviate pain associated with sciatic nerve palsy post-THA, making it preferable to conservative management [10]. In this case, a cautious approach was adopted due to the patient's diffuse lipomatosis and unclear anatomy around the gluteal region. After a week of conservative treatment without significant improvement, neurolysis was performed. Unfortunately, full sensory and functional recovery of the patient's left lower limb was not achieved.

## 4 Conclusion

Diffuse lipomatosis can cause limb deformity, complicate the management of other conditions, such as DDH, and increase the risk of surgical complications like sciatic nerve entrapment. Neurological symptoms in the lower limbs should prompt consideration of sciatic nerve issues, particularly in patients with anatomical abnormalities. Changing body positions may help prevent nerve entrapment. Once sciatic nerve entrapment is diagnosed, early intervention with neurolysis is recommended to prevent permanent neurological damage.

## Author contribution

Cai Jian, MD (Writing – original draft; Writing – review & editing)

Zhou Guanming, BS (Conceptualization; Resources; Supervision)

Guan Mingqiang, MD (Writing – review & editing)

Zeng Huiliang, MM (Investigation)

## Consent

Written informed consent was obtained from the patient for publication of this case report and accompanying images. A copy of the written consent is available for review by the Editor-in-Chief of this journal on request.

## Ethical approval

This study has been approved by the Medical Ethics Committee of Foshan Hospital of Traditional Chinese Medicine. Approval Number: KY[2024]122.

## Guarantor

Zhou Guanming.

## Research registration number

None.

## Funding

This study received funding from Foshan City 14th Five Year Plan Medical High level Key Specialized Construction Project.

## Conflict of interest statement

The authors declared no conflict of interest.
